# Exploring mHealth app utilization for diabetes self-management: survey insights from a northern district in Malaysia

**DOI:** 10.1186/s12889-024-21056-w

**Published:** 2024-12-19

**Authors:** Premaa Supramaniam, Ying-Shan Beh, Suria Junus, Philip Rajan Devesahayam

**Affiliations:** 1 Clinical Research Centre, Hospital Raja Permaisuri Bainun, Institute for Clinical Research, National Institute of Health, Ministry of Health Malaysia, Ipoh, Perak, Malaysia; 2 Outpatient Pharmacy Unit, Greentown Health Clinic, Ministry of Health, Perak, Malaysia

**Keywords:** mHealth, Applications, Diabetes mellitus, Questionnaire, Experience, Challenges

## Abstract

**Background:**

Mobile health applications (mHealth apps) offer potential benefits for improving diabetes management, such as better glucose monitoring and patient engagement, but their widespread adoption faces challenges, including privacy concerns and user adherence. This research investigates mHealth app usage among patients living with diabetes in Kinta District, Perak, exploring experiences, challenges and patient perceptions regarding diabetes management using mHealth apps.

**Methodology:**

A cross-sectional community survey was conducted in September till November 2020 across nine government health clinics focusing on diabetes mellitus (Type 1 or Type 2) patients, aged 18 years and older, receiving Diabetes Medication Adherence Counseling (DMTAC) services and able to use smart devices. A self-developed questionnaire with four sections was used to gather demographic information, explore mHealth apps usage and understand both users and non-users’ experiences and perceptions. The questionnaire was tested through cognitive debriefing, translated into Malay, pre-tested and finalized by the expert committee. The questionnaire was digitally implemented using Google^®^ Form and QR code. After obtaining informed consent, data collection was performed by the trained DMTAC pharmacists. Statistical analyses involved descriptive and inferential analyses.

**Results:**

The study analyzed the engagement of 295 patients living with diabetes with mHealth apps. Females (54.9%), of Malay ethnicity (58.3%) and with a mean age of 53.8 years (SD: 12.38) constituted the majority. Diabetes duration had a median of 6 years (IQR: 3.0, 10.0) with prevalent comorbidities like hypertension (58.0%) and dyslipidemia (42.7%). Most patients were employed (44.7%) and their primary source of diabetes management information was through healthcare providers (92.5%). Despite the high app use for social interaction, only 13.6% used mHealth apps for disease management. Users were influenced by social media (65.0%) and favored for wellness apps and disease monitoring. Users perceived the mHealth app as useful (97.5%), yet faced challenges over the app initiation, charges and data security. Non-users cited lack of awareness (70.2%), struggled with app startup (22.4%) and preference for conventional healthcare visits (22.0%). In multivariable analysis, longer diabetes duration reduced mHealth app usage (*p* = 0.046), while multimorbidity increased the likelihood (*p* = 0.001). Awareness of the availability of health apps significantly influenced the usage of mHealth apps (*p* < 0.001).

**Conclusion:**

The findings highlight the underutilization of mHealth apps for diabetes management despite their perceived usefulness. Challenges faced by users and non-users underscore the need for more awareness, thus encourage widespread acceptance and usage of mHealth apps in diabetes care.

## Introduction

Diabetes mellitus (DM) represents a significant and growing healthcare burden worldwide, particularly in Malaysia [1–3]. The National Health and Morbidity Survey 2015 reported a concerning prevalence of 17.5% (3.5 million individuals) among Malaysian adults aged 18 and older, with projections suggesting an increase to 31.3% (7 million individuals) by 2025 [4, 5]. This escalating prevalence underscores the urgent need for effective diabetes management strategies.

Various interventions have been implemented to enhance diabetes management, including medication adherence assessments, identification and management of drug-related problems, medication counselling, monitoring of clinical outcomes and diabetes education. Diabetes Medication Therapy Adherence Clinic (DMTAC) is a service where pharmacists, with physicians’ support, help diabetic patients improve medication adherence and glycaemic control through assessments, counselling and diabetes education over at least eight follow-up visits. The service resulted in significant improvements in glycaemic control, as evidenced by reductions in HbA1c (1.0–1.73%) and fasting blood glucose levels (2.65 mmol/L) among patients with Type 2 diabetes [6]. These outcomes highlight the effectiveness of structured interventions in managing diabetes.

In addition to existing interventions integrated into diabetes care, mobile health applications (mHealth apps) have emerged as adjuncts to diabetes self-management. Utilization of these applications for self-management in countries such as Saudi Arabia, Norway and Australia has demonstrated patient preferences for features such as continuous glucose monitoring, blood glucose measurement, data recording of physical activity, medication adherence, dietary tracking and communication with healthcare professionals [7–11]. Furthermore, mHealth apps have been used by patients with diabetes to aid in glycaemic control and blood pressure monitoring [12, 13]. Among mHealth users, personal preferences indicate a desire for enhanced engagement with healthcare providers [14–16], visual aids for educational materials [9–11, 15] and reminder functionalities [15, 16]. Generally, mHealth apps designed for diabetes patients are developed to provide specialized tools and functionalities for effective diabetes management [17].

Despite the promising potential of mHealth apps, several challenges and limitations hinder their widespread adoption for diabetes management. Barriers to utilization include time-consuming data entry requirements, which can discourage consistent use [8, 9, 14, 15], a lack of awareness about available applications that could assist with self-management [8, 10, 11], and difficulties navigating the technology due to poor user interface design or limited digital literacy [8–10]. Additionally, factors such as behavioral intention, perceived ease of use, and personal attitudes toward technology play a significant role in determining the effectiveness and continued use of these apps for disease management [18]. Privacy concerns and data security issues also deter some patients from using health apps to track medical information. There is a gap in understanding how to select and customize diabetes apps to meet the diverse needs of patients, as existing solutions often lack features tailored to individual preferences and specific clinical requirements.

Furthermore, as the integration of mHealth applications into structured diabetes care is not yet formalized, assessing the current status of voluntary mHealth app usage among patients with diabetes is crucial. Understanding how patients independently utilize these technologies can provide insights into their potential benefits and limitations for disease self-management. Recommendations for mobile technologies, including diabetes-specific mHealth applications, emphasize the importance of active involvement by both patients and healthcare professionals to maximize their effectiveness [19]. Evidence suggests that mHealth apps can support various aspects of diabetes management, such as monitoring blood glucose levels, medication adherence, lifestyle modifications and patient-healthcare interaction [12, 13]. Integrating mHealth apps into structured programs DMTAC has the potential to enhance patient engagement, optimize clinical outcomes and provide a more personalized approach to diabetes care.

This study aims to explore the usage of mHealth apps among patients living with diabetes in the Kinta District, located in the northern state of Perak, Malaysia. The research seeks to assess the current status of self-initiated mHealth app usage among patients with diabetes mellitus in the district, with the objective of establishing a foundational understanding of their voluntary usage, choices, challenges and perceptions regarding health applications for diabetes management. Ultimately, this research aims to provide insights that could inform future interventions and enhance the effectiveness of mHealth apps in improving diabetes care.

## Materials and methods

### Study design

A cross-sectional study was conducted by administering a community survey through self-administered questionnaires in government health clinics in Kinta District, Perak. The Kinta District in the State of Perak, covers an area of 1305 km, with a population density of 685/km^2^ with three government hospitals and 36 government clinics, alongside with private healthcare facilities as of year 2021 [20]. All nine government health clinics with Diabetes Medication Adherence Counseling (DMTAC) services were identified to be the recruiting sites for the study. Data was collected prospectively between September till November 2020.

### Survey respondents

The survey respondents consisted of patients living with diabetes who were enrolled in the DMTAC at government healthcare clinics in the Kinta district and were aged 18 years or older. Participants for the survey were recruited by inviting all eligible patients with diabetes who met the inclusion criteria during the three-month period from September to November 2020. Participants were required to be able to converse in either Malay or English. Foreigners and patients deemed physically or mentally unable to engage in a conversation or self-administer the questionnaire were excluded from the study.

### Sample size and sampling technique

Although evidence regarding the use of mHealth apps for self-management among Malaysian patients living with diabetes is limited, a study reported a 36.6% prevalence of diabetes patients using mHealth apps for management in Saudi Arabia [8]. To ensure a 0.5 precision at a 0.05 significance level, the study required a minimum of 357 patients living with diabetes. The distribution of survey respondents across healthcare clinics in the district was estimated based on the proportion of active DMTAC patients in 2019. Eligible patients were recruited using a convenient sampling technique for the study.

### Study instrument and data collection process

The study instrument was a self-developed questionnaire, created based on quantitative and qualitative published literature findings focusing on the use of mHealth apps for diabetes self-management [7, 9–11, 14, 15, 17–24]. The questionnaire consisted of four sections. Part I of the questionnaire gathered demographic details, including participants’ age, gender, ethnicity, marital status, education level, occupation, medical history, duration of diabetes, current medication, and sources of referrals for diabetes-related issues. Part II addressed general questions about the use of smart devices, with a particular focus on assessing participants’ baseline awareness of mHealth apps that could aid in managing health issues, especially diabetes. Part III covered various aspects of mHealth app usage, including the types of apps used, their features and functionalities, obstacles encountered, and the overall satisfaction experienced by users. Lastly, Part IV investigated the reasons for non-use among participants who did not engage with mHealth apps. The questionnaire primarily utilized multiple-choice and Likert scale questions.

To enhance item clarity, cognitive debriefing was conducted with 10 patients living with diabetes at a selected health clinic. Feedback and suggestions were incorporated, simplifying complex terminology while maintaining the original content. The questionnaire was then translated into Malay following established international standards, including forward and backward translations by independent translators [25–28]. A pre-test of the Malay version was conducted with an additional 10 patients living with diabetes to further refine the translated items.

Although formal validation was not performed, as there were no latent constructs to measure, the final English and Malay versions were extensively reviewed by an expert panel comprising endocrinologists, public health experts, and pharmacists to ensure content accuracy. The finalized questionnaire was then adapted into an electronic version with a QR code linking to both the English and Malay versions to facilitate participant access.

Trained data collectors, comprising pharmacists from the Diabetes Medication Therapy Adherence Clinic (DMTAC), were involved in the data collection process. To ensure voluntary participation and minimize attrition rates, the appointed pharmacists served as the resident pharmacists in charge of the DMTAC at their respective healthcare clinics.

Eligible patients were approached, and after obtaining written informed consent from those who agreed to participate, a QR code was provided to enable them to self-administer the questionnaire using their smartphones. Data collectors were available to assist participants as needed. Scheduled data quality monitoring was conducted by the study investigators to ensure the completeness and accuracy of the collected data.

### Statistical analysis

The questions in the questionnaire were analysed on an item-by-item basis, rather than assessing latent concepts. For descriptive analysis, normally distributed continuous data was presented with descriptive summaries of mean and standard deviation (SD) while non-normally distributed data was presented using median and inter-quartile range (IQR). Categorical data was presented using frequency and percentages and 95% confidence interval (CI) where applicable.

Binary logistic regression was used to test the relationship between mHealth apps usage and the associating factors. Variable selection for multivariable analysis was made based on *p*-value of 0.25 and less. Odds ratio (OR) was presented with 95% CI. *P*-value of less than 0.05 was considered statistically significant. Hosmer-Lemeshow test, multicollinearity issues between the independent factors and explained variance (R^2^) were investigated to judge the variables fitness in the multivariable model. Backward likelihood ratio (LR) method was used for the multivariable analysis. Data was re-coded and analysed using SPSS version 20.0 (IBM Corp. Released 2011. IBM SPSS Statistics for Windows, Version 20.0, Armonk, NY: IBM Corp).

## Results

### Response rate

A total of 333 potential patients with diabetes were approached across nine healthcare clinics in the Kinta District, Perak. Among these individuals, 295 patients (82.6%) consented to participate in the study. Of the consenting participants, 106 patients (31.8%) preferred the English version of the questionnaire, while 227 patients (68.2%) preferred the Malay version.

### Survey respondents’ distribution

The study predominantly consisted of female participants (54.9%), with the majority being of Malay ethnicity (58.3%), married (87.8%) and possessing a secondary level of education (53.2%). The mean age of participants was 53.8 years (SD: 12.38). Most patients were employed (44.7%), followed by retirees (22.7%) (Table 1).

**Table 1:** Demographic and medical profiles of the diabetic patients in Kinta District (*n* = 295)

Variables	*n* (%)
***Demographic profiles***	
Gender	
Female	162 (54.9)
Male	133 (45.2)
Age (years) [mean (SD)]	53.8 (12.38)
Ethnicity	
Malay	172 (58.3)
Chinese	55 (18.6)
Indian	68 (23.1)
Marital status	
Married	258 (87.8)
Unmarried	23 (7.8)
Divorced / Widow / Widower	14 (4.7)
Highest Education Level	
Primary	59 (20.0)
Secondary	157 (53.2)
Tertiary	69 (23.4)
No formal education	10 (3.4)
Occupation	
Employed	132 (44.7)
Unemployed	96 (32.5)
Retired	67 (22.7)
***Medical profiles***	
Comorbidities (multiple responses)	
Diabetes mellitus	295 (100.0)
Hypertension	171 (58.0)
Dyslipidemia	126 (42.7)
Others*	22 (7.5)
Duration of diabetes (years) [median, IQR]	6.0 (3.0, 10.0)
Diabetes medication regimes	
Oral medication only	169 (57.3)
Both oral and insulin	115 (39.0)
Insulin therapy only	11 (3.7)
Referral sources for diabetes management (multiple responses)	
Healthcare providers	273 (92.5)
Family and friends	78 (26.4)
Online resources	57 (19.3)
Mobile applications	9 (3.1)

*Asthma, heart-, lung-, kidney-related disease, gout, gastric, cancer and thyroid problems; SD-standard deviation, IQR-inter-quartile range presented with 25th and 75th percentiles

The median duration of diabetes among patients was 6 years (IQR: 3.0, 10.0). A majority of these patients also presented with concomitant conditions such as hypertension (58.0%), dyslipidemia (42.7%) and other comorbidities (7.5%), such as asthma, organ-specific diseases, gout, gastric issues, cancer and thyroid problems. Approximately 96.3% of patients were receiving monotherapy, primarily with oral diabetes medications. Regarding diabetes management, the primary source of information for respondents was healthcare providers (HCPs) (92.5%), followed by advice from family and friends (26.4%). Only a small proportion (3.1%) relied on mobile applications for diabetes-related concerns (Table 1).

### mHealth apps use for diabetes management

**Fig. 1:**
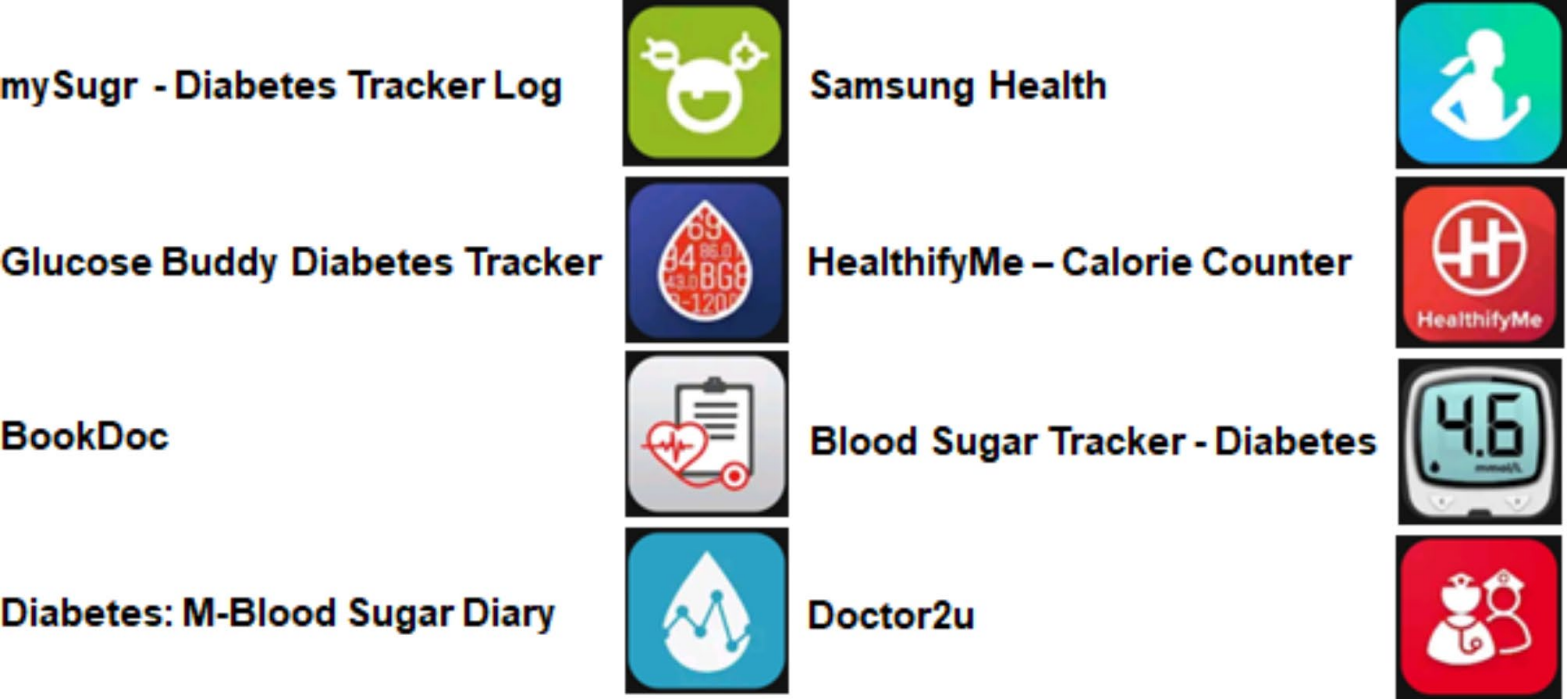
Self-initiated mHealth apps usage by patients with diabetes, Perak [29–36]

Smart devices, including smartphones and tablets, were used for an average of 3.0 h daily (IQR: 1.0, 5.0). The majority of patients reported downloading applications primarily for social interaction and transportation purposes (89.5%), followed by accessing news and updates (33.6%) and engaging in entertainment through games (26.8%). Approximately 23.1% of survey respondents used apps related to health and lifestyles, while 21.7% employed apps for educational and business purposes. Surprisingly, more than half of the respondents (65.1%) were unaware of the availability of mHealth applications. Only 13.6% of patients with diabetes reported having used at least one mHealth app for disease management [Table 2]. Among the mHealth apps used by the respondents were *mySugr* [29], *Glucose Buddy* [30], *BookDoc* [31], *Diabetes: M* [32], *Doctor2u* [33], *Blood Sugar Tracker-Diabetes* [34], *HealthifyMe* [35] and *Samsung Health* [36] [Fig. 1].

**Table 2:** Usage of smart devices by diabetic patients in Kinta District (*n* = 295)

Variables	*n* (%)
Duration of smart devices usage per day (hours) [median, IQR]	3.0 (1.0, 5.0)
Types of mobile applications downloaded (multiple responses)	
Social interaction and transportation	264 (89.5)
News & updates	99 (33.6)
Games	79 (26.8)
Health and lifestyles	67 (22.7)
Educational materials	41 (13.9)
Business-related	23 (7.8)
Awareness of availability of health apps	
Unaware	192 (65.1)
Aware	103 (34.9)
Used at least ONE mHealth apps	
No	255 (86.4)
Yes*	40 (13.6)

*mySugr, Glucose Buddy, BookDoc, Diabetes: M, Doctor2u, Blood Sugar Tracker – Diabetes, HealthifyMe and Samsung Health

### Features of mHealth apps described by the users

Among the mHealth app users (*n* = 40), the majority were motivated by social media (65.0%), followed by influence from close contacts such as family and friends (35.0%). More than half of the users employed these apps primarily for physical fitness, weight management, diet and nutrition. For disease monitoring and management, the apps offered features such as vital signs tracking (42.5%), blood glucose monitoring (37.5%), foot pressure monitoring (10.0%) and medication reminders (25.0%). Additionally, some users utilized the apps for communication with HCP and support in smoking cessation (Table 3).

**Table 3:** mHealth apps usage for diabetes management (*n* = 40)

Variables	*n* (%)
mHealth apps introduced by (multiple responses)	
Social media	26 (65.0)
Family member & friends	14 (35.0)
HCP	9 (22.5)
Types of mHealth app features (multiple responses)	
Physical fitness	27 (67.5)
Weight management	24 (60.0)
Diet and nutrition	22 (55.0)
Vital signs monitoring	17 (42.5)
Blood glucose monitoring	15 (37.5)
Medication reminders	10 (25.0)
Communication with HCP	9 (22.5)
Foot pressure monitoring	4 (10.0)
Smoking cessation	3 (7.5)
mHealth app documentation functionalities (multiple responses)	
Weight measurements	19 (47.5)
Exercise records	17 (42.5)
Diet & nutrition records	13 (32.5)
Blood glucose measurement	12 (30.0)
Medication records	10 (25.0)
Blood pressure measurement	8 (20.0)
Preferred additional mHealth app features (multiple responses)	
Reading materials on diabetes education	31 (77.5)
Visual aids on diabetes education	18 (45.0)
Online interaction with HCP	16 (40.0)
Peer discussion on diabetes management	13 (32.5)
Frequency of mHealth apps usage	
When educational information is required	19 (47.5)
Scheduled usage (daily/weekly/monthly)	14 (35.0)
Occasionally	7 (17.5)

HCP-Health care provider

Health apps offer various features for tracking and recording health-related information, with weight measurement (47.6%) and exercise logs (42.5%) being the most frequently used among users. Additionally, features supporting diabetes management, such as accessing educational materials (77.5%), using visual aids for learning (45.0%), engaging in online interactions with healthcare providers (40.0%) and participating in peer discussions on diabetes management (32.5%), were highly preferred. Most users accessed these apps on an as-needed basis or according to a specific schedule (Table 3).

### Experiences and perceptions of using mHealth apps for diabetes management

The majority of users perceived mHealth apps as highly beneficial for managing their diabetes, with 97.5% acknowledging their positive impact on disease management. Despite this, 75.0% of respondents still preferred assistance from healthcare professionals (HCPs). Regarding ease of use, 65.0% found navigating the downloaded apps straightforward, while 30.0% felt they required some form of training. However, 40.0% reported initial difficulties in using the apps due to a lack of prior knowledge. Additional concerns were noted, including the cost of apps, data security and privacy issues and the tedious nature of record-keeping (Table 4).

**Table 4:** Users’ experiences and personal perception for using mHealth apps for diabetes management (*n* = 40)

Variables	*n* (%)
Perceived usefulness of mHealth apps	
Useful	39 (97.5)
Not useful	1 (2.5)
Perceived satisfaction	
Satisfied with HCP assistance	30 (75.0)
Satisfied without HCP assistance	3 (7.5)
Not satisfied and requires HCP assistance	2 (5.0)
Unable to define satisfaction level	5 (12.5)
Difficulty level of mHealth apps navigation	
Easy	26 (65.0)
Requires training	12 (30.0)
Difficult	2 (5.0)
Hurdles with mHealth apps (multiple response)	
Unsure how to start	16 (40.0)
Consumes too much time for recordings	15 (37.5)
Fee for apps installation	14 (35.0)
Worried about data security	11 (27.5)
Online access of mHealth database by HCP will ease diabetes management	
Strongly agree	15 (37.5)
Agree	17 (42.5)
Not sure	8 (20.0)
Peer-coaching via mHealth apps could assist diabetes management	
Strongly agree	11 (27.5)
Agree	9 (22.5)
Not sure	14 (35.0)
Disagree	6 (15.0)
HCP-patient communication via mHealth apps could assist diabetes management	
Strongly agree	11 (27.5)
Agree	15 (37.5)
Not sure	13 (32.5)
Disagree	1 (2.5)

HCP-Health care provider

Several suggestions emerged for improving the functionality of mHealth apps in diabetes management. A substantial majority (80.0%) agreed that allowing HCPs to access patients’ mHealth app databases online would enhance patient care. Around half of the respondents supported incorporating peer discussion features for sharing experiences and problem-solving related to diabetes care, although 15.0% disagreed with this idea. Additionally, 65.0% expressed support for using mHealth apps as a tool for patient-HCP communication (Table 4).

### Reasons for non-usage of mHealth apps

An assessment investigating the reasons behind non-usage of mHealth apps among non-users (*n* = 255) revealed that a majority (70.2%) were unaware of the availability of such apps that could aid in disease management, while 22.0% preferred conventional healthcare facility visits. Additional barriers included difficulties with app installation and navigation, as well as concerns about data security, privacy and associated costs. Despite these challenges, nearly half of the respondents indicated a willingness to consider using mHealth apps for future diabetes management. Their preferences leaned towards exploring apps focused on fitness and dietary management (58.8%), vital signs and glucose monitoring (54.5%) and medication tracking (40.8%) (Table 5).

**Table 5:** Reasons for not using mHealth apps and future consideration (*n* = 255)

Variables	*n* (%)
Reasons for not using mHealth apps (multiple response)	
Unaware about mHealth apps	179 (70.2)
Hurdles with apps installation and navigation	57 (22.4)
Preferred conventional visits to healthcare facilities	56 (22.0)
Fees for apps	23 (9.0)
Worried for data protection & privacy	19 (7.5)
No interest	11 (4.3)
Future consideration for health monitoring apps features (multiple response)	
Fitness & dietary management	150 (58.8)
Vital signs and glucose monitoring	139 (54.5)
Medication tracking	104 (40.8)
No consideration	11 (4.3)
Future consideration for using mHealth apps for diabetes management	
Yes	125 (49.0)
No	45 (17.6)
I don’t know	85 (33.3)

*Associating factors of the mHealth apps usage among patients living with diabetes* Univariable logistic regression analysis showed that age, the presence of multimorbidity, seeking advice from family, friends and online materials, various types of downloaded apps and awareness about mHealth were significantly associated among mHealth users (*p* < 0.05) (Table 6).

**Table 6:** Univariable and multivariable analyses of the associating factors of mHealth apps use among patients living with diabetes (*n* = 295)

Variables	mHealth apps users (*n* = 40), n (%)	mHealth apps non-users (*n* = 255), n (%)	Crude OR (95% CI)	*p*-value	Adjusted OR (95% CI)	*p*-value
***Demographic profiles***						
Gender						
Female	21 (52.5)	141 (55.3)	1.00 (reference)	-		
Male	19 (47.5)	114 (44.7)	0.89 (0.45, 1.74)	0.741		
Age (years) [mean (SD)]	50.1 (11.36)	54.4 (12.45)	0.97 (0.95, 0.99)	***0.043***		
Ethnicity						
Malay	28 (70.0)	144 (56.5)	1.46 (0.63, 3.38)	0.380		
Chinese	4 (10.0)	51 (20.0)	0.59 (0.17, 2.07)	0.408		
Indian	8 (20.0)	60 (23.5)	1.00 (reference)	-		
Marital status						
Married	32 (80.0)	226 (88.6)	0.85 (0.18, 3.97)	0.836		
Unmarried	6 (15.0)	17 (6.7)	2.12 (0.36, 12.34)	0.404		
Divorced / Widow / Widower	2 (5.0)	12 (4.7)	1.00 (reference)	-		
Highest Education Level						
Primary	3 (7.5)	56 (22.0)	0.48 (0.05, 5.16)	0.546		
Secondary	19 (47.5)	138 (54.1)	1.24 (0.15, 10.33)	0.843		
Tertiary	17 (42.5)	52 (20.4)	2.94 (0.35, 24.94)	0.322		
No formal education	1 (2.5)	9 (3.5)	1.00 (reference)	-		
Occupation						
Employed	27 (67.5)	105 (41.2)	2.20 (0.91, 5.37)	0.082	1.36 (0.41, 4.49)	0.618
Unemployed	6 (15.0)	90 (35.3)	0.57 (0.18, 1.78)	0.335	0.37 (0.08, 1.60)	0.181
Retired	7 (17.5)	60 (23.5)	1.00 (reference)	-	1.00 (reference)	-
***Medical profiles***						
Duration of diabetes (years) [median, IQR]	5.0 (2.3, 9.8)	6.0 (3.0, 10.0)	0.95 (0.90, 1.01)	0.100	0.92 (0.85, 0.99)	0.046
Comorbidities (multiple responses)						
Hypertension	26 (65.0)	145 (56.9)	1.41 (0.70, 2.82)	0.334		
Dyslipidaemia	14 (35.0)	112 (43.9)	0.69 (0.34, 1.38)	0.291		
Others*	8 (20.0)	14 (5.5)	4.30 (1.68, 11.06)	***0.002***	12.05 (2.96, 49.11)	***0.001***
Diabetes medication regimes						
Oral medication only	20 (50.0)	149 (58.4)	0.68 (0.34, 1.34)	0.262		
Insulin therapy only	1 (2.5)	10 (3.9)	0.51 (0.06, 4.18)	0.527		
Both oral and insulin	19 (47.5)	96 (37.6)	1.00 (reference)	-		
References for diabetes management (multiple responses)						
Healthcare providers	38 (95.0)	235 (92.2)	1.62 (0.36, 7.20)	0.528		
Family and friends	18 (45.0)	60 (23.5)	2.66 (1.34, 5.29)	***0.005***		
Online resources	16 (40.0)	41 (16.1)	3.48 (1.70, 7.12)	***0.001***		
Mobile applications	3 (7.5)	6 (2.4)	3.37 (0.81, 14.04)	0.096		
***Usage of smart devices***						
Duration of smart devices usage per day (hours) [mean (SD)]	3.9 (2.15)	3.5 (3.53)	1.03 (0.94, 1.13)	0.505		
Types of mobile applications downloaded (multiple responses)						
Social interaction and transportation	34 (85.0)	230 (90.2)	0.62 (0.24, 1.61)	0.323		
News & updates	18 (45.0)	81 (31.8)	1.76 (0.89, 3.46)	0.102		
Games	16 (40.0)	63 (24.7)	2.03 (1.02, 4.07)	***0.045***		
Health and lifestyles	26 (65.0)	41 (16.1)	9.69 (4.67, 20.13)	***< 0.001***	12.82 (4.63, 35.45)	***< 0.001***
Educational materials	13 (32.5)	28 (11.0)	3.90 (1.81, 8.43)	***0.001***		
Business-related	6 (15.0)	17 (6.7)	2.47 (0.91, 6.70)	0.076		
Awareness of availability of health apps						
Unaware	3 (7.5)	189 (74.1)	1.00 (reference)	-	1.00	-
Aware	37 (92.5)	66 (25.9)	35.32 (10.54, 118.38)	***< 0.001***	53.09 (12.58, 224.09)	***< 0.001***

*Asthma, heart-, lung-, kidney-related disease, gout, gastric, cancer and thyroid problems; OR-Odds ratio, CI-confidence interval, SD-Standard deviation, IQR-inter-quartile range; Binary Logistic Regression was used for the analysis; Included variables with *p* < 0.25 for multivariable analysis; Model fit with R^2^ = 0.597, Backward LR method was used, Hosmer-Lemeshow *p* = 0.714, Overall classification = 90.8%

In the multivariable analysis, a longer duration of diabetes was associated with a lower likelihood of mHealth app usage [AOR: 0.92 (95% CI: 0.85, 0.99), *p* = 0.046]. In contrast, patients with additional health conditions, such as asthma, organ-specific diseases, gout, gastric issues, cancer and thyroid problems, in addition to diabetes were significantly more likely to use mHealth apps [AOR: 12.05 (95% CI: 2.96, 49.11), *p* = 0.001]. Furthermore, individuals who had previously downloaded health and lifestyle apps [AOR: 12.82 (95% CI: 4.63, 35.45), *p* < 0.001], as well as those who were aware of mHealth apps availability [AOR: 53.09 (95% CI: 12.58, 224.09), *p* < 0.001], showed a higher likelihood of utilizing mHealth apps (Table 6).

## Discussion

### Key findings

This study investigated the use of mHealth apps among patients with diabetes in a central district of Perak, Malaysia. While smartphone and smart device ownership was widespread, only a small proportion of patients approximately one in seven (13.6%) utilized mHealth apps for diabetes management. This utilization rate is considerably lower than global estimates, which report mHealth app usage among diabetic populations ranging from 33.3 to 48.8% [7, 8, 11, 15]. The most commonly preferred app functionalities identified by study respondents included features related to disease monitoring and management, such as vital signs tracking, blood glucose monitoring, foot pressure monitoring, medication reminders, and communication with healthcare providers.

### Preferences for features in mHealth apps

The integration of diabetes-specific functionalities within mHealth apps, such as real-time blood glucose and foot pressure monitoring, has revolutionized diabetes management. These functionalities serve as indispensable tools, enabling users to actively monitor vital health metrics and engage in continuous tracking, interventions and enhanced adherence to treatment regimens, thereby significantly improving health outcomes [7, 8, 11, 15]. The blood glucose monitoring feature facilitates frequent glucose level assessments, allowing for timely interventions and lifestyle adjustments that benefit Type II diabetic patients [37–42]. Similarly, foot pressure apps not only provide foot care education but also enhance self-management skills by enabling the early detection of potential complications, such as diabetic foot ulcers, through meticulous foot checks and visual aids [43]. This proactive approach is instrumental in fostering effective foot care practices [38, 44–47].

Additionally, medication reminder features within mHealth apps serve as essential tools for adherence, ensuring the timely administration of prescribed medications and minimizing the risk of missed doses, thereby substantially enhancing treatment efficacy and overall health management [15, 48]. These applications are equipped with a suite of functionalities, including scheduling, tracking options, short messaging services (SMS) and tailored visual aids, comprehensively catering to diverse adherence levels and various user needs for effective self-management strategies [49–51]. Notification reminders can alleviate forgetfulness and reduce medication errors, particularly among patients undergoing prolonged treatments and the elderly [52].

The findings of the present study highlight significant user engagement, with 77.5% of mHealth app users accessing diabetes-related reading materials and 45.0% utilizing visual aids for disease education. These mobile apps serve as platforms that replace traditional paper-based resources, providing a streamlined alternative to navigating various websites for information. By offering convenient access to validated and up-to-date materials tailored to individual needs, these apps cater to users seeking comprehensive knowledge and insights into diabetes management and lifestyle adjustments [53]. Research among Type II diabetes patients has revealed a correlation between higher health literacy and improved diabetes knowledge, enhanced glucose control and reduced smoking rates, underlining the crucial role of health literacy in effective diabetes self-management [54]. Equipped with comprehensive information, individuals can make informed decisions regarding diet, exercise, medication adherence and overall lifestyle adjustments, ultimately leading to an improved quality of life [39, 55]. Similarly, recognizing therapeutic goals is pivotal, as it has the potential to enhance adherence to medications, dietary adjustments and physical activity regimens [56–58].

The integration of enhanced features that promote peer support through online discussions among patients living with diabetes within mHealth apps facilitates the sharing of experiences and the exchange of peer-based suggestions. The present study indicated that half of the app users agreed that peer coaching via mHealth apps could assist in diabetes management. This platform enables the online sharing of personal insights and strategies for diabetes management. Peer support fosters a supportive network, allowing individuals to gain diverse perspectives, learn from others’ experiences and receive practical tips and encouragement [59–61]. This empowerment equips individuals with practical, real-world modifications for disease management [62]. Internet and mobile interventions, coupled with peer support, not only facilitate easy access to essential knowledge but also enhance self-management behaviours, medication adherence and treatment adherence [59, 60, 63, 64].

Additionally, communication features that reflect a healthy patient-HCP relationship via mHealth apps serve as another tool for enhancing diabetes self-management. This feature emphasizes direct, seamless interaction between patients and healthcare professionals, transcending geographical barriers and enabling real-time consultations, advice and health updates [65]. The utility of this communication channel extends to improved healthcare accessibility, especially for individuals in remote locations or those facing mobility constraints, ensuring continuous support and guidance from HCP. Its contribution to disease self-management lies in empowering patients through immediate access to guidance on medication adjustments, dietary concerns or emergent complications, thereby facilitating timely interventions and informed decision-making [47, 66, 67]. In a study conducted by Kim et al. (2020) exploring patient-centred communication (PCC) strategies within an mHealth-based Diabetes Prevention Program (DPP) for older adults, researchers found that coaches provided consistent support by engaging users in regular and spontaneous in-app chats. This interactive chat system significantly enhanced health awareness, facilitated the review of electronic medical records, and triggered behavioural changes among patients [68].

### Challenges in mHealth apps usage

In contrast, the present study found that nearly three out of ten mHealth app users reported concerns about data security. Similar concerns regarding the safety and confidentiality of patient medical information have been documented globally [15, 69–71]. Data security and privacy issues arise from the sensitive nature of health-related information stored in these apps, leading to fears of unauthorized access, data breaches and potential misuse of personal health data. These concerns can significantly impede the widespread adoption of mHealth technologies, as individuals often prioritize protecting their private information over the convenience offered by digital health tools. It is essential to address these security concerns through strong data encryption, strict compliance with data protection regulations, a robust consent process and transparent privacy policies [69, 72–74]. Additionally, initiatives aimed at educating users about app security features and their rights regarding data privacy are crucial for fostering trust and confidence in mHealth technologies [75, 76]. Such efforts can help alleviate apprehensions and support the integration of digital solutions into routine diabetes care.

The present study identified factors associated with mHealth app usage among patients living with diabetes, revealing an inverse relationship between the duration of diabetes and app utilization. This inverse correlation suggests that individuals with a longer duration of diabetes tend to engage less with mHealth apps. Newly diagnosed patients exhibited increased activity within mHealth apps across various modules, such as nutrition, fitness, medication and glucose monitoring. However, they faced challenges in sustaining commitment when manual data input was required [77]. This trend is be influenced by habituation to conventional management methods, resulting in reluctance to embrace new technological interventions [78]. Furthermore, long-term patients often have established routines, which pose challenges in integrating new technologies into their existing management approaches [78, 79]. Overcoming these challenges may necessitate the intrinsic design of user-friendly interfaces, personalized guidance during app navigation and targeted education that emphasizes the long-term benefits of mHealth apps.

### Factors influencing mHealth app usage

The present study revealed positive associations between mHealth app utilization and the presence of multimorbidity, awareness of mHealth apps and the use of other common health and lifestyle apps. Individuals managing multiple health conditions, in addition to diabetes, derive significant value from using mHealth apps for decision-making regarding their overall well-being, as these tools facilitate the holistic management of their diverse health concerns [80]. Users actively engaging with various health and lifestyle apps readily embrace mHealth apps as complementary tools for managing their health. This positive correlation underscores the potential for integrated health management solutions and suggests that individuals already invested in health-centric technologies are more inclined to adopt mHealth apps as an extension of their proactive approach to overall well-being. Furthermore, a systematic search in app stores indicates that apps designed for patients with multimorbidity, recorded with higher score on the Mobile App Rating Scale (MARS) and the App Behavior Change Scale (ABACUS). Self-monitoring of physiological parameters such as blood glucose tracking, being the most common feature [81].

### Study limitations and future directions

Despite the valuable insights garnered with low attrition rate in the study, this study is limited by the cross-sectional design with convenient sampling of study respondents which provides a snapshot of mHealth app utilization among patients living with diabetes, limited in capturing longitudinal changes or causality. Additionally, the geographic focus on the Kinta District of Perak state, might limit the generalizability of findings to broader regional context. Furthermore, the reliance on self-reported data introduces potential for recall bias and subjective interpretations, impacting the accuracy of responses and perceptions. The self-developed questionnaire was not subjected to formal validation, as it was primarily designed to collect descriptive data rather than measure latent constructs.

Future studies should consider conducting a comprehensive validation process, including assessments of reliability and validity, to enhance the robustness and generalizability of the findings. Future research should consider longitudinal or interventional study designs to track changes in app usage patterns over time, assess causal relationships between app utilization and health outcomes, explore differences in app preferences based on the needs and life experiences of Type 1 and Type 2 diabetes patients and evaluate healthcare providers’ preparedness and confidence in effectively supporting patients’ use of mHealth apps for self-management. A broader geographical coverage encompassing diverse demographics and stratified cultural contexts would enhance the understanding of varied adoption patterns.

### Implication of the study

The findings from this study have significant implications for the implementation of mHealth apps aimed at improving diabetes management in Malaysia. The low adoption rate of mHealth apps among patients living with diabetes underscores the critical need for targeted interventions to enhance user engagement. Identifying specific functionalities that users prefer, such as real-time monitoring of vital signs, medication reminders and educational resources, can inform the development of user-friendly and relevant mHealth apps that cater to the needs of this population. Additionally, addressing concerns regarding data security and privacy is paramount, implementing robust security measures and transparent privacy policies can foster trust and encourage greater acceptance of digital health tools.

The positive associations found between mHealth app utilization and multimorbidity suggest that patients with complex health needs may benefit from integrated health management solutions. This highlights the potential for mHealth apps to function not only as tools for diabetes management but also as platforms for comprehensive health support. Making the use of mHealth apps a mandatory addition to routine patient care could ensure that all patients have access to these essential tools, ultimately improving clinical outcomes. Furthermore, healthcare providers should effectively communicate the benefits and functionalities of mHealth apps, bridging the gap between traditional management practices and innovative digital solutions.

## Conclusion

The study among patients living with diabetes in Kinta District provided crucial insights into mHealth app usage. Despite a median diabetes duration of 6 years and widespread smart device usage, only 13.6% utilized mHealth apps for diabetes self-management. Features such as vital signs, blood glucose and foot pressure monitoring and medication reminders were highly preferred. The integration of diabetes-specific functionalities, coupled with patient-HCP communication and peer support, showed promising prospects for enhancing diabetes self-management and improving overall quality of life. However, the low adoption rates underscore the necessity for improved intrinsic designs with improved security measures and targeted interventions to encourage widespread acceptance and usage of mHealth apps in diabetes care.

## Data Availability

Data and materials used in the study for this manuscript are available from the corresponding author upon request. The translated version of questionnaire and the dataset generated for the current study are available from the corresponding author on reasonable request.

## Abbreviations

ABACUS: App Behavior Change Scale
AOR: Adjusted odds ratio
CI: Confidence interval
DM: Diabetes mellitus
DMTAC: Diabetes Medication Adherence Counseling
DPP: Diabetes Prevention Program
HCP: Healthcare provider
IQR: Inter-quartile range
LR: Likelihood ratio
MARS: Mobile App Rating Scale
OR: Odds ratio
PCC: Patient-centered communication
PIS: Participant Information Sheet
SD: Standard deviation
SMS: Short-messaging services
